# Schmallenberg virus antibody persistence in adult cattle after natural infection and decay of maternal antibodies in calves

**DOI:** 10.1186/1746-6148-10-103

**Published:** 2014-05-01

**Authors:** Armin RW Elbers, Norbert Stockhofe-Zurwieden, Wim HM van der Poel

**Affiliations:** 1 Department of Epidemiology, Crisis organisation and Diagnostics, Central Veterinary Institute, part of Wageningen UR, PO Box 65, NL-8200AB Lelystad, Netherlands; 2 Department of Infection Biology, Central Veterinary Institute, part of Wageningen UR, PO Box 65, NL-8200AB Lelystad, Netherlands; 3 Department of Virology, Central Veterinary Institute, part of Wageningen UR, NL-8200AB, NL-8200AB Lelystad, Netherlands

**Keywords:** Schmallenberg virus, Cattle, Dairy cows, Immunity, Maternal antibodies, Virus-neutralizing antibodies

## Abstract

**Background:**

Schmallenberg virus (SBV) has swept through the major part of Europe in the period 2011–2013. A vaccine against SBV has been developed and may be a possible preventive instrument against infection. Presently, there is no data available to refute the assumption that natural SBV infection results in long-term immunity. In that respect, it is of interest to know how long (protecting) virus-neutralizing antibodies are present in naturally infected animals. New-born calves acquire passive immunity from their dams by ingestion and absorption of antibodies present in colostrum, which can block the production of serum antibodies when vaccine is administered to calves with maternally derived antibodies. In that respect, it is useful to know how long it takes for maternal antibodies against SBV to disappear in young animals born from infected dams.

**Results:**

Longitudinal *w*hole-herd serological monitoring using virus neutralization test (VNT) indicated that 80% of adult dairy cows still had measurable antibodies against SBV at least 24 months after the estimated introduction of the virus into the herd. Median ^2^Log VNT titer of the adult dairy cows (≥1 year) dropped from 8.6 to 5.6 in a period of 17 months. Median ^2^Log VNT maternal antibodies titers of calves sampled within 30 days after birth was 8. Calves lost their maternally-derived antibodies after 5–6 months. There was a definite positive relationship between the VNT titer of the dam and the VNT titer of the corresponding calf (age ≤ 30 days) of dam-calf combinations sampled on the same day: the higher the VNT titer of the dam, the higher the VNT titer (maternal antibodies) of the calf.

**Conclusions:**

Our field data support the assumption that natural SBV infection in adult cows results in persistence of specific antibodies for at least two years. Based on the observed decay of maternally-derived antibodies in calves, it is presumed safe to vaccinate calves against SBV at an age of approximately 6 months.

## Background

Schmallenberg virus (SBV), a novel *Orthobunyavirus*, has swept through the major part of Europe in the period 2011–2013, reaching as far North as Finland, Turkey in the East, and Spain in the South [1,2]. A serological survey of blood samples, collected in the period November 2011 – January 2012, from a representative sample of cattle in the Netherlands indicated a high seroprevalence of antibodies against SBV [3]. So far, the impression is that the clinical impact of the disease on ruminant livestock is limited [1]. A vaccine against SBV has been developed [4] and may be a possible preventive instrument against infection. Presently, there is no data available to refute the assumption that natural SBV infection results in long-term immunity [1], as was seen earlier with natural infection of cattle with bluetongue virus serotype 8 [5]. In that respect, it is of interest to know how long (protecting) virus-neutralizing antibodies are present in naturally infected animals. Newborn calves acquire passive immunity from their dams by ingestion and absorption of antibodies present in colostrum. The estimated duration and benefit of this passively derived humoral immunity can vary greatly depending on the colostrum production (quantity and quality) and the quantity of antibody ingested and absorbed [6]. Passive immunity can block the production of serum antibodies when vaccine is administered to calves with maternally-derived antibodies [7]. In that respect, it is useful to know how long it takes for maternal antibodies against SBV to disappear in young animals born from infected dams. To examine the development and the course of neutralizing-antibodies against SBV over time, in a dairy herd in the eastern part of the Netherlands where SBV RNA was detected in a high proportion of *Culicoides* caught in the autumn of 2011 [8], all animals were blood-sampled five times in a 17-month period, and tested for SBV specific neutralizing antibodies.

## Results and discussion

**Figure 1:**
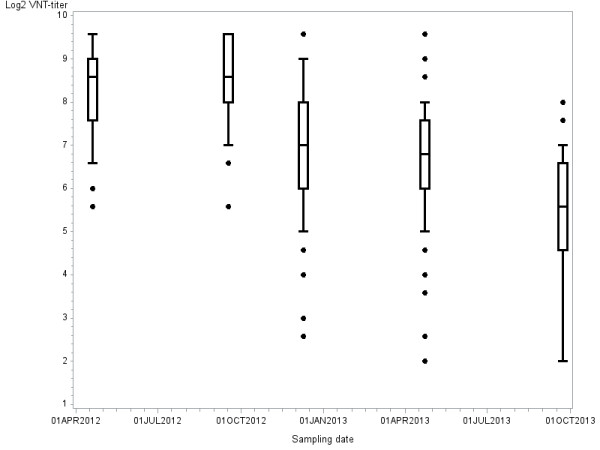
**Distribution of virus neutralization test (VNT) antibody titers (**^**2**^**Log-scale) against Schmallenberg virus in dairy cattle ≥ 1 year of age by date of sampling, summarized by a box and whisker plot (the central line in the box plot indicates the median of the data, while the edges of the box indicate the 25th and 75th percentiles; extending from the box are whiskers, the top whisker expands to the 90th percentile and the bottom whisker to the 10th percentile; beyond the whiskers are observations that are relatively far from the median).**

At the 1st sampling (19 April 2012), all adult cows (≥1year of age) tested seropositive, only four calves (6 months of age) tested seronegative. Seventeen months later at the fifth sampling (23 September 2013), 80% of the adult cows still tested seropositive, while only 9% of the cows < 1year of age tested seropositive (Table 1). Median ^2^Log VNT titer of the adult cows dropped from 8.6 (range: 5.5 – 9.5) to 5.6 (range: 2 – 8) in a period of 17 months (Figure 1). It can be assumed that the adult cows became infected around the time that SBV RNA was detected for the first time in *Culicoides* biting midges caught at this dairy farm on 14 September 2011 [8]. This means that at least 24months after natural infection, animals most likely were protected against re-infection.

**Table 1:** **Distribution of cows with a **^**2**^**Log virus neutralization test titer ≥ 3 by sampling date and age category**

**Sampling date**	**Total number of cows**	**Number of cows ≥ 1year**	**Number of cows ≥ 1year with **^**2**^**Log VNT titer ≥ 3**	**Number of cows < 1 year**	**Number of cows < 1year with **^**2**^**Log VNT titer ≥ 3**
19 Apr 2012	108	87	87 (100%)	21	17 (81%)
17 Sept 2012	108	89	89 (100%)	19	19 (100%)
9 Dec 2012	110	89	88 (99%)	21	16 (76%)
23 Apr 2013	116	86	84 (98%)	30	15 (50%)
23 Sept 2013	109	87	70 (80%)	22	2 (9%)

Eleven heifers seroconverted (seronegative in April 2012 and high VNT-titers (^2^Log VNT titer ≥ 6) in September 2012) and one dairy cow seroconverted between the September and December 2012 samplings. The low rate of seroconversions matched with a significantly lower (six times) proportion of SBV-infected *Culicoides* observed in 2012 compared to 2011 [9], and it can be assumed that there was a much lower level of SBV-circulation in the area in 2012 compared to 2011.

**Figure 2:**
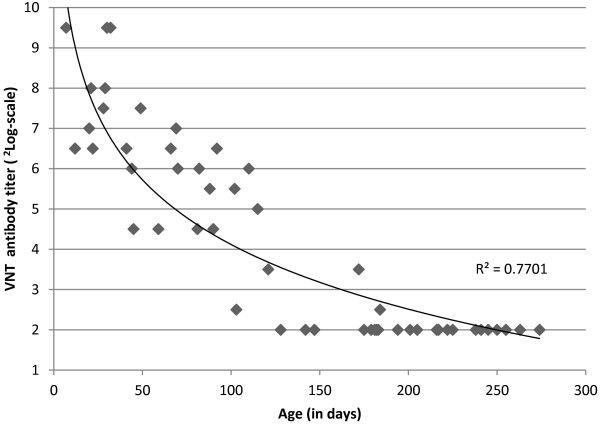
Virus neutralization test (VNT) antibody titers against Schmallenberg virus of 25 calves by age at sampling (based on 2–3 samplings per calf).

Calves that were bled ≤ 30 days after birth had a median ^2^Log VNT titer of 8 (range: 6.5 - 9.5) and became seronegative (^2^Log VNT titer < 3) within 5–6 months (Figure 2). The presence of maternally-derived antibodies may hamper the effective response to vaccination. Therefore, it is important to consider the age at which the calves lose their maternal antibody, previous to starting a vaccination campaign. There are sparse reports on the course of decay of maternal antibodies against *Orthobunyaviruses* in ruminants. Tsutsui et al. [10] showed that dairy calves lost their maternally derived antibodies against Akabane virus at an age of approximately 4 months, while Grimstad et al. [11] showed that young white-tailed deer lost their maternal antibodies against Jamestown Canyon virus at an age of 5–6 months. Our results seem to be in range with the above-mentioned studies. To achieve an effective response to vaccination it can be advised to vaccinate calves at an age of at least 6 months.

**Figure 3:**
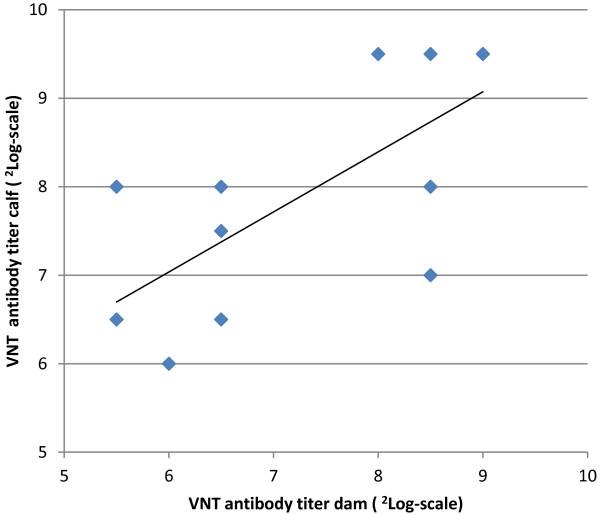
**Relationship between virus neutralization test antibody titer (**^**2**^**Log-scale) against Schmallenberg virus of calves and their dams (sampled the same day).** Age of calves was ≤ 30 days.

There was a definite positive relationship (correlation coefficient: 0.73, significance level: < 0.01) between the VNT titer of the dam and the VNT titer of the corresponding calf (age ≤ 30 days) of 13 dam-calf combinations sampled on the same day (Figure 3): the higher the VNT titer of the dam, the higher the VNT titer (maternal antibodies) of the calf.

## Conclusions

Our field data support the assumption that natural SBV infection in adult cows results in persistence of specific antibodies for at least two years. Based on the observed decay of maternally-derived antibodies in calves, it is presumed safe to vaccinate calves against SBV at an age of approximately 6months.

## Methods

From all animals of a dairy herd in the eastern part of the Netherlands blood samples were taken five times in a 17-month period to be able to examine persistence and titers of neutralizing-antibodies against SBV over time in adult cattle and calves.

Blood samples were obtained in the context of non-experimental clinical veterinary practices for diagnostic purposes and procedures followed were performed in accordance with the Dutch national as well as European Union animal experiment regulations.

The dairy herd consisted of 60 milking cows, 50 heifers and calves; no animals from other herds were purchased onto the farm, a few older adult cows were sold including all the male calves born. This dairy herd is the only location in the Netherlands where *Culicoides* monitoring was executed continuously and where SBV RNA was detected in *Culicoides* caught in 2011 [8] and 2012 [9]. No SBV clinical signs were observed in any cattle of the dairy herd at the end of 2011 or the beginning of 2012. All animals of the herd were blood-sampled, for the first time on 19 April 2012 after retrospective detection of SBV RNA in the *Culicoides* collection caught in 2011 [8].

A total of three calves were stillborn during the one-year study period on this dairy, but all without the characteristic malformations observed after SBV infection, which was confirmed by gross pathology and all tissue samples of these calves tested SBV-negative by RT-PCR.

Blood samples were tested for antibodies using a virus neutralization test (VNT) [12], with some small modifications: dilutions tested started at 1:4 and ended at 1:512. All samples were tested in duplicate. Titers were determined using the Reed-Münch method [13] and expressed on a ^2^Logarithm-scale. Distribution of VNT-titers by sampling date was summarized with a box and whisker plot [14] in Figure 2: the central line in the box plot indicates the median of the data, while the edges of the box indicate the first and third quartiles (that is, the 25th and 75th percentiles); extending from the box are whiskers, the top whisker expands to the 90th percentile and the bottom whisker to the 10th percentile; beyond the whiskers are observations that are relatively far from the median.

All animals of the herd were again blood sampled on 17 September 2012 (5 months after 1st sampling), 9 December 2012 (8 months after 1st sampling), 23 April 2013 (12 months after 1st sampling) and 23 September 2013 (17 months after 1st sampling).

## Competing interests

The authors declare they have no competing interests.

## Authors’ contributions

AE designed the study, carried out the study in the field, performed the analysis and drafted the manuscript. WP contributed to the analysis of the data. NS carried out pathology on stillborn calves of the dairy herd. NS and WP commented on the draft manuscript. All authors read and approved the final manuscript.
